# Abnormal plasma DNA profiles in early ovarian cancer using a non-invasive prenatal testing platform: implications for cancer screening

**DOI:** 10.1186/s12916-016-0667-6

**Published:** 2016-08-24

**Authors:** Paul A. Cohen, Nicola Flowers, Stephen Tong, Natalie Hannan, Mark D. Pertile, Lisa Hui

**Affiliations:** 1 St John of God Hospital Bendat Family Comprehensive Cancer Centre, Subiaco, Perth, Western Australia Australia; 2 Department of Perinatal Medicine, Mercy Hospital for Women, Heidelberg, Melbourne, Victoria Australia; 3 Victorian Clinical Genetics Services, Parkville, Victoria Australia; 4 Translational Obstetrics Group, Mercy Hospital for Women, Heidelberg, Melbourne, Victoria Australia; 5 Department of Obstetrics and Gynaecology, University of Melbourne, Parkville, Victoria Australia; 6 Murdoch Children’s Research Institute, Parkville, Victoria Australia; 7 School of Women’s and Infants’ Health, University of Western Australia, Subiaco, Perth, Western Australia Australia; 8 Department of Paediatrics, University of Melbourne, Parkville, Victoria Australia; 9 Institute for Health Research, University of Notre Dame Australia, Fremantle, Western Australia Australia

**Keywords:** Non-invasive prenatal testing, High-grade serous carcinoma, Ovarian cancer screening, Circulating tumor DNA, Low coverage sequencing, Copy number variations, Genomic profiling, Liquid biopsy

## Abstract

**Background:**

Non-invasive prenatal testing (NIPT) identifies fetal aneuploidy by sequencing cell-free DNA in the maternal plasma. Pre-symptomatic maternal malignancies have been incidentally detected during NIPT based on abnormal genomic profiles. This low coverage sequencing approach could have potential for ovarian cancer screening in the non-pregnant population. Our objective was to investigate whether plasma DNA sequencing with a clinical whole genome NIPT platform can detect early- and late-stage high-grade serous ovarian carcinomas (HGSOC).

**Methods:**

This is a case control study of prospectively-collected biobank samples comprising preoperative plasma from 32 women with HGSOC (16 ‘early cancer’ (FIGO I–II) and 16 ‘advanced cancer’ (FIGO III–IV)) and 32 benign controls. Plasma DNA from cases and controls were sequenced using a commercial NIPT platform and chromosome dosage measured.

Sequencing data were blindly analyzed with two methods: (1) Subchromosomal changes were called using an open source algorithm WISECONDOR (WIthin-SamplE COpy Number aberration DetectOR). Genomic gains or losses ≥ 15 Mb were prespecified as “screen positive” calls, and mapped to recurrent copy number variations reported in an ovarian cancer genome atlas. (2) Selected whole chromosome gains or losses were reported using the routine NIPT pipeline for fetal aneuploidy.

**Results:**

We detected 13/32 cancer cases using the subchromosomal analysis (sensitivity 40.6 %, 95 % CI, 23.7–59.4 %), including 6/16 early and 7/16 advanced HGSOC cases. Two of 32 benign controls had subchromosomal gains ≥ 15 Mb (specificity 93.8 %, 95 % CI, 79.2–99.2 %). Twelve of the 13 true positive cancer cases exhibited specific recurrent changes reported in HGSOC tumors. The NIPT pipeline resulted in one “monosomy 18” call from the cancer group, and two “monosomy X” calls in the controls.

**Conclusions:**

Low coverage plasma DNA sequencing used for prenatal testing detected 40.6 % of all HGSOC, including 38 % of early stage cases. Our findings demonstrate the potential of a high throughput sequencing platform to screen for early HGSOC in plasma based on characteristic multiple segmental chromosome gains and losses. The performance of this approach may be further improved by refining bioinformatics algorithms and targeting selected cancer copy number variations.

**Electronic supplementary material:**

The online version of this article (doi:10.1186/s12916-016-0667-6) contains supplementary material, which is available to authorized users.

## Background

The detection and monitoring of specific cancer mutations by sequencing circulating DNA holds much promise, but has yet to be widely translated into clinical care. In contrast, sequencing plasma DNA during pregnancy to detect fetal chromosomal abnormalities (non-invasive prenatal testing, NIPT) has been rapidly implemented globally due to its high accuracy and proven clinical validity [1].

Circulating DNA of tumor origin can interfere with NIPT performance and produce abnormal genomic profiles that suggest occult malignancy in pregnant women [2]. Amant et al. [3] recently reported the pre-symptomatic identification of cancer in three pregnant women undergoing NIPT, suggesting that genomic profiling for copy number variations (CNVs) may be a feasible approach for cancer screening. However, the sensitivity and specificity of clinical NIPT platforms for cancer remains unknown.

Ovarian cancer is the leading cause of gynecologic cancer-related deaths in developed countries [4] and there is a pressing need for an effective screening test [5, 6]. High-grade serous ovarian cancer (HGSOC) accounts for most deaths from the disease [7] and demonstrates a marked chromosomal instability [8]. We hypothesized that these tumor-derived chromosome abnormalities would be detectable in the plasma of HGSOC patients collected prior to primary surgery. The aims of this study were to investigate whether a clinical NIPT platform could detect HGSOC in the non-pregnant population based on an abnormal plasma DNA profile, and to compare the detection rates for early and advanced stage HGSOC.

## Methods

We performed a case control study of 64 plasma samples obtained from the Western Australia Gynecologic Oncology Biospecimen Bank. These were prospectively collected between January 2013 and August 2015 with informed consent from patients prior to undergoing surgery. Ethical approval was granted for this study.

The 32 cancer cases comprised 16 women with International Federation of Gynecology and Obstetrics (FIGO) stage I and II HGSOC (‘early cancer’), and 16 women with FIGO stages III and IV HGSOC (‘advanced cancer’). The control group included women with benign gynecologic disease undergoing surgery (*n* = 24), or germline *BRCA1* and *BRCA2* mutation carriers without evidence of malignancy who were undergoing risk-reduction surgery (*n* = 8).

DNA libraries, prepared from cell-free DNA extracted from plasma, were sequenced on a commercial whole genome NIPT platform using the standard workflow employed for aneuploidy screening (percept™ prenatal test, Victorian Clinical Genetics Services, Parkville VIC Australia, based on Illumina’s verifi™ NIPT methodology [2]). Each research sample was sequenced alongside 14 clinical samples, with 36-cycle single-end sequencing on an Illumina NextSeq500. The read depth was low coverage at 0.2× to 0.3× based on 18–28 M × 36 bp single end reads. Laboratory and analysis staff were blinded to the case/control allocation of samples. Two types of data analyses were performed.

1. We used the open source algorithm WISECONDOR (WIthin-SamplE COpy Number aberration DetectOR) to detect whole chromosome and subchromosomal abnormalities not identifiable by the standard NIPT pipeline [9]. Segmental changes > 15 Mb were prespecified as abnormal calls (“positive cancer screen”).
2. We also analyzed the sequence data using the routine clinical percept™ pipeline, developed to detect fetal aneuploidy for chromosomes 21, 18, 13, X, and Y.

Paired tumor DNA was unavailable to correlate with plasma sequencing data. We therefore compared the results of the WISECONDOR analysis with somatic CNVs reported in the Integrated Genomic Analyses of Ovarian Carcinoma (IGAOC) derived from 489 HGSOC tumor genomes by The Cancer Genome Atlas Research Network [8]. Our data were examined for recurrent regional aberrations affecting extended chromosome regions that were reported as statistically significant by the IGAOC (8 gains and 22 losses).

## Results

We detected 6/16 early stage and 7/16 advanced stage HGSOC cases using the WISECONDOR analysis, giving an overall detection rate of 13/32 (sensitivity 40.6 %, 95 % CI, 23.7–59.4 %). There were two false positive calls in the control group (specificity 93.8 %, 95 % CI, 79.2–99.2 %) (Table 1).

**Table 1:** Sequencing copy number variation calls using percept™ pipeline and WISECONDOR algorithm

Group	Stage	WISECONDOR call		Percept™ call			Total
		Abnormal	Normal	Low risk	No call	High risk	
Early HGSOC	FIGO I–II	6	10	14	2	0	16
Advanced HGSOC	FIGO III–IV	7	9	12	3	1^a^	16
Benign	N/A	2	30	29	1	2^b^	32

^a^Monosomy 18 call

^b^Two monosomy X calls

HGSOC, high grade serous ovarian carcinoma; FIGO, International Federation of Gynecology and Obstetrics

**Fig. 1:**
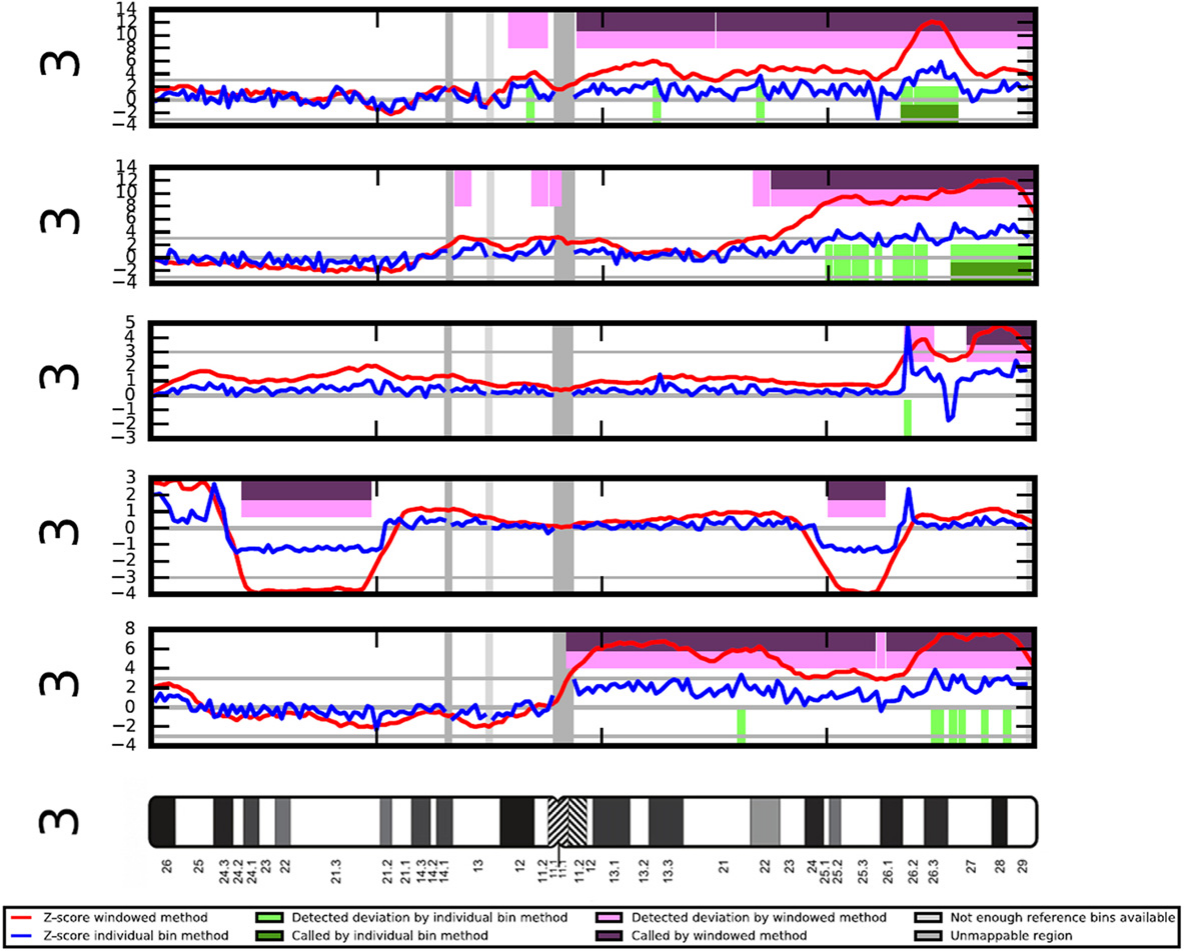
WISECONDOR plots of sequenced cfDNA showing copy number variations of chromosome 3 in the plasma of five subjects with high-grade serous ovarian carcinomas. From top, Subject 1 diagnosed with a stage 2C, Subject 2 stage 2C, Subject 3 stage 4, Subject 4 stage 3C, Subject 5 stage 3C, and an Ideogram of chromosome 3. Y axis of plots depicts Z-score; *red* and *blue* lines are Z-score plotted by windowed and individual bin methods, respectively. *Pink* and *purple* bars indicate deviation detected by windowed method or called by windowed method, respectively [12]. Subjects 1, 2, 3, and 5 show whole arm and/or segmental gains of chromosome 3q. Subject 4 shows segmental copy number losses within chromosome 3p and 3q

Table 2 presents the specific CNVs detected in the 13 true positive cancer cases and the two false positive controls. Twelve of the 13 true positive cancer calls had a CNV that was reported in The Cancer Genome Atlas Network as statistically significant (FDR *q* value < 0.25) at high frequency (>50 % of tumors). The most common DNA amplifications observed in the 13 true positive calls affected chromosome arms 3q (*n* = 5), 8q (*n* = 7), 20q (*n* = 4), and 12p (*n* = 3). The most common DNA losses were seen on chromosome arms 5q (*n* = 3), 8p (*n* = 3), 13q (*n* = 4), and 15q (*n* = 3). Figure 1 shows the WISECONDOR plots of sequenced cfDNA showing copy number variations of chromosome 3 in the plasma of five subjects with high-grade serous ovarian carcinomas.

**Table 2:** “Screen positive” copy number variations (CNVs) in 13 cancer cases and two controls mapped to reported gains and losses in the Integrated Genomic Analysis of Ovarian Cancer (IGAOC) study [11]

Subject number	Age (years)	Study group	FIGO Stage	Percept™ call for aneuploidy	Detected CNVs ≥ 15 Mb mapped according to IGAOC^a^		
					Highly specific	Moderately specific	Non-specific
1	76	Early stage cancer	2C	No call	Chr 3q gain Chr 12p gain Chr 20q terminal gain Chr 5q segmental loss Chr 8p loss Chr 9p loss		Chr 5p gain Chr 7q segmental loss
2	65	Early stage cancer	2C	No call	Chr 3q terminal gain Chr 20 gain Chr 4q loss Chr 7p loss Chr 13q segmental loss Chr 15q segmental loss	Chr 6p gain	Chr 2q interstitial gain Chr 18q segmental gain
3	48	Early stage cancer	1C	Low risk	Chr 12p terminal gain		
4	71	Early stage cancer	2C	Low risk	Chr 3q interstitial gain Chr 8q gain		
5	38	Early stage cancer	1C	Low risk	Chr 8q terminal gain		Chr 3p terminal gain
6	47	Early stage cancer	2A	Low risk	Chr 8q terminal gain		
7	54	Advanced stage cancer	4	No call	Chr 3q terminal gain Chr 8 gain Chr 5q loss Chr 13 loss Chr 15 loss Chr 17 loss Chr 18 loss Chr 22 loss	Chr 14 loss	Chr 5p gain Chr 9p gain
8	57	Advanced stage cancer	3B	Low risk	Chr 8q terminal gain Chr 8p terminal loss	Chr 1q interstitial gain Chr 6p gain	Chr 1p interstitial gain Chr 11q segmental gain
9	60	Advanced stage cancer	3A1	Low risk	Chr 20 gain		
10	83	Advanced stage cancer	3A	Low risk			Chr 11q interstitial gain
11	33	Advanced stage cancer	3C	No call	Chr 8q terminal gain Chr 12p terminal gain Chr 4q segmental loss Chr 5q interstitial loss Chr 6q terminal loss Chr 8p loss Chr 9p terminal loss Chr 13 segmental loss Chr 15 segmental loss Chr 17q segmental loss Chr 22 loss	Chr 6p segmental gains Chr 7q segmental gains	Chr 1p segmental gains Chr 2 segmental gains Chr 5p gain Chr 11q interstitial gain Chr 18q segmental gain Chr 1p segmental loss Chr 10p loss Chr 11q terminal loss Chr 21 loss
12	58	Advanced stage cancer	3C	No call	Chr 3q gain Chr 4p loss Chr 9q loss Chr 13 loss	Chr 1q gain Chr 6p gain Chr 7q terminal gain Chr 11p loss	Chr 5p loss Chr 7p terminal loss Chr 10p gain Chr 18 gain
13	66	Advanced stage cancer	3C	Monosomy 18	Chr 20q gain Chr 8q terminal segmental gain		
14	44	Benign control	NA	Low risk	Chr 20q segmental gain		
15	53	Benign control	NA	Low risk	Chr 20q gain		

^a^CNVs are categorized according to IGAOC analysis [8]. The IGAOC found 8 significantly gained chromosome arms (5 present in > 50 % of tumor samples), and 22 significantly deleted chromosome arms (18 present in > 50 %). We used the following definitions: highly specific CNV, statistically significant gain or loss (*q* value < 0.25) with frequency in > 50 %; Moderately specific CNV, statistically significant gain or loss (*q* value < 0.25) with frequency in < 50 %; non-specific CNV, gain or loss with *q* value > 0.25

The percept™ pipeline resulted in one “monosomy 18” call from the cancer group, and two “monosomy X” calls in the controls (Table 2). In five cancer cases and one control case, the pipeline failed to produce a result because of unexpected profiles on normalizing chromosomes.

A post hoc analysis of our results showed that many smaller focal aberrations identified by the IGAOC were also present in the “screen positive” cancer cases. Most of the cancer cases had multiple focal changes, whereas none of the benign controls, including the two false positive calls, had more than one focal change (Additional file 1).

The two false positives in the control groups in the WISECONDOR analysis had single segmental gains on 20q. The clinical history of these controls included a benign fallopian tube cyst in a patient with endometriosis and a hemorrhagic follicular cyst in a patient with a prior history of breast ductal carcinoma in situ which had been completely excised prior to plasma collection. Both patients were alive with no clinical evidence of malignant or systemic disease at the time of writing.

## Discussion

In this proof of concept study, low coverage plasma DNA sequencing and analysis for chromosomal CNVs ≥ 15 Mb detected 40 % of HGSOC. Surprisingly, we detected similar proportions of early and advanced stage HGSOC cancers with this approach. This finding was unexpected because one would assume a higher detection rate in the advanced stage cases, given the lower tumor bulk of early disease. This suggests that the detection of ovarian tumor CNVs in plasma is not directly related to cancer stage; other biological factors such as fractional concentration of tumor DNA in plasma, tumor genetic heterogeneity, vascularity, and cell turnover may also be important influences on detection rates.

A limitation of our study was the inability to correlate the plasma sequencing data with paired tumor DNA due to the absence of suitable archived specimens. However, the principle that tumor DNA is detectable in plasma using NIPT sequencing platforms has been previously established [2, 3]. Furthermore, the majority of genomic aberrations detected in our cases included common imbalances previously reported in a cohort of 489 HGSOC specimens [8], supporting our assumption that the DNA aberrations detected in plasma originated from ovarian tumors.

Prior “liquid biopsy” studies in ovarian cancer have relied on the identification of tumor-specific mutations in advanced disease and the postoperative monitoring of patient-specific mutations in plasma via deep sequencing [10, 11]. Our results are notable for demonstrating that it is possible to detect early stage ovarian cancer in the absence of patient-specific tumor DNA using an existing low coverage sequencing platform. Thus, high throughput whole genome plasma sequencing, with or without the addition of other biomarkers, is an exciting avenue for future studies of cancer screening. It may have utility as a cost-effective method of monitoring high risk patients for whom tumor tissue is unavailable, such as presymptomatic *BRCA1/2* mutation carriers, or to assess the preoperative risk of malignancy in patients presenting with ovarian masses.

Potential reasons for the false positive WISECONDOR results in the two controls include technical issues with the archived plasma samples or reference chromosome set. The two “monosomy X” calls in the NIPT pipeline in the controls (aged 43 and 54 years) might be explained by normal age-related X chromosome loss [12] or low grade mosaicism [13]. It is plausible that, with larger cohorts, algorithms could be devised that increase test specificity. Further work is also required to assess the technical issues with archived plasma samples and to develop the clinical potential of this approach.

## Conclusions

A low coverage plasma DNA sequencing protocol used in a high throughput prenatal screening platform detected more than one in three women with early stage ovarian cancer based on common segmental chromosome gains and losses. Further refinement of this approach may have utility for future studies of ovarian cancer screening.

## Additional file

Additional file 1: CNVs >15 Mb in the Western Australia Biospecimen Bank Dataset, CNVs reported in an ovarian cancer genome atlas, and coordinates of overlapping regions. (XLS 52 kb)

## Abbreviations

CNV: copy number variation
HGSOC: high grade serous ovarian carcinoma
NIPT: non-invasive prenatal testing
WISECONDOR: within sample copy number aberration detector
